# Muscle miRNAome shows suppression of chronic inflammatory miRNAs with both prednisone and vamorolone

**DOI:** 10.1152/physiolgenomics.00134.2017

**Published:** 2018-06-08

**Authors:** Alyson A. Fiorillo, Christopher B. Tully, Jesse M. Damsker, Kanneboyina Nagaraju, Eric P. Hoffman, Christopher R. Heier

**Affiliations:** ^1^Center for Genetic Medicine Research, Children’s National Medical Center, Washington, District of Columbia; ^2^Department of Genomics and Precision Medicine, George Washington University School of Medicine and Health Sciences, Washington, District of Columbia; ^3^ReveraGen BioPharma, Incorporated, Rockville, Maryland; ^4^School of Pharmacy and Pharmaceutical Sciences, Binghamton University, State University of New York, Binghamton, New York

**Keywords:** Duchenne muscular dystrophy, inflammation, miRNA, muscle, steroids

## Abstract

Corticosteroids are highly prescribed and effective anti-inflammatory drugs but the burden of side effects with chronic use significantly detracts from patient quality of life, particularly in children. Developing safer steroids amenable to long-term use is an important goal for treatment of chronic inflammatory diseases such as Duchenne muscular dystrophy (DMD). We have developed vamorolone (VBP15), a first-in-class dissociative glucocorticoid receptor (GR) ligand that shows the anti-inflammatory efficacy of corticosteroids without key steroid side effects in animal models. miRNAs are increasingly recognized as key regulators of inflammatory responses. To define effects of prednisolone and vamorolone on the muscle miRNAome, we performed a preclinical discovery study in the *mdx* mouse model of DMD. miRNAs associated with inflammation were highly elevated in *mdx* muscle. Both vamorolone and prednisolone returned these toward wild-type levels (miR-142-5p, miR-142-3p, miR-146a, miR-301a, miR-324-3p, miR-455-5p, miR-455-3p, miR-497, miR-652). Effects of vamorolone were largely limited to reduction of proinflammatory miRNAs. In contrast, prednisolone activated a separate group of miRNAs associated with steroid side effects and a noncoding RNA cluster homologous to human chromosome 14q32. Effects were validated for inflammatory miRNAs in a second, independent preclinical study. For the anti-inflammatory miRNA signature, bioinformatic analyses showed all of these miRNAs are directly regulated by, or in turn activate, the inflammatory transcription factor NF-κB. Moving forward miR-146a and miR-142 are of particular interest as biomarkers or novel drug targets. These data validate NF-κB signaling as a target of dissociative GR-ligand efficacy in vivo and provide new insight into miRNA signaling in chronic inflammation.

## INTRODUCTION

Duchenne muscular dystrophy (DMD) is a lethal genetic disease with pediatric onset and is characterized by progressive muscle degeneration with chronic inflammation. The current DMD standard of care is chronic treatment with high-dose corticosteroids (prednisone, deflazacort). Prednisone and deflazacort both increase DMD patient strength, prolong ambulation, and reduce scoliosis, however their long-term use is associated with many side effects that negatively impact patient quality of life (1). Side effects noted as particular concerns to children with DMD and their families include stunted growth, bone fragility, mood disturbances, and weight gain. Accordingly, the development of effective drugs that are safer than corticosteroids is an important goal for DMD and other chronic disorders currently treated with steroids.

To develop an improved drug, it is important to dissect how prednisone works at the molecular level. The drug target of prednisone is the glucocorticoid receptor (GR). Once activated by prednisone the GR exerts its effects by *1*) binding to other proteins to affect their functions, and *2*) moving into the nucleus where it directly binds to DNA promoters to affect gene expression through glucocorticoid response elements (GREs). Many anti-inflammatory effects of prednisone are believed to be caused by GR protein interactions, where the GR inhibits the inflammatory transcription factor NF-κB (58). However, some have hypothesized that prednisone efficacy in DMD is mediated through other functions, either by the direct actions of the GR in binding to DNA to activate GREs in gene promoters (57) or by gross physiological effects such as growth stunting (20). Moving forward, it is important to determine which of these GR properties can be selectively activated, as well as which GR properties are expendable versus which properties are essential for efficacy in treating DMD and other chronic disorders. Vamorolone (VBP15) is a first-in-class dissociative steroid that binds the GR with high affinity (23). Data to date on individual gene targets suggest that vamorolone/GR complexes retain many protein-binding activities of prednisone/GR complexes (e.g., NF-κB inhibition), but vamorolone/GR complexes do not activate gene targets as do prednisone/GR complexes (e.g., GRE transactivation). Thus, vamorolone loses transactivation (gene transcription) activities associated with side effect profiles of corticosteroids, while maintaining anti-inflammatory activities associated with efficacy.

Recently, miRNAs have emerged as a promising new class of biomarkers and therapeutic targets. Specific proinflammatory microRNAs are becoming increasingly implicated in chronic inflammatory states, as reviewed in Ref. 64. miRNAs are relatively stable, are highly conserved across species, and because miRNAs are not translated into a protein product, their expression can be directly correlated to function. Typically, miRNAs exert their functions by binding to the 3′ untranslated region of mRNA and either inhibiting their translation or promoting mRNA decay, thereby downregulating corresponding protein expression (80). Their stability and conservation across species contribute to their appeal as biomarkers (33, 46). miRNAs are also becoming increasingly attractive therapeutic targets (31).

Here, we analyze expression of the miRNAome in *mdx* dystrophic muscle to dissect molecular signatures that drive effects of contrasting drug treatments at the genomic level in vivo. We are developing vamorolone (VBP15) as a dissociative GR ligand and have previously reported that it shows efficacy similar to prednisone in the *mdx* mouse model of DMD, in the absence of traditional steroid side effects in the *mdx* mouse (23). Here we utilize a more holistic, -omic approach to study the larger scale molecular effects of chronic prednisolone and vamorolone treatment in vivo. This approach enables us to dissect the molecular pathways that are shared versus differentiated for these two GR ligands, which share efficacy but are differentiated in safety profiles at the organismal level (23). We identify a key set of nine miRNAs that are all elevated by muscular dystrophy disease and return toward healthy wild-type levels upon treatment with both drugs. All nine miRNAs are directly activated by or in turn activate the inflammatory transcription factor NF-κB. These data provide a key group of miRNAs for the development of novel biomarkers and therapies, while also validating chronic inflammatory NF-κB signaling pathways as a target of dissociative steroid efficacy in vivo.

## MATERIALS AND METHODS

### 

#### Animal care.

All mouse studies were performed in adherence to the NIH Guide for the Care and Use of Laboratory Animals. All experiments were conducted according to protocols that were within the guidelines and approval of the Institutional Animal Care and Use Committee of Children’s National Medical Center. All *mdx* (C57BL/10ScSn-Dmd<*mdx*>/J) and wild-type control (C57BL/10ScSnJ) mice were obtained from The Jackson Laboratory (Bar Harbor, ME).

#### Drug dosing and mouse muscle samples.

Archival muscle samples (diaphragm) from two separate preclinical studies were obtained, with each trial showing a significant benefit from both prednisolone and vamorolone drug treatments (23). Prednisolone was used because it is the active form of prednisone, the current DMD standard of care. The first “discovery set” of diaphragm muscles was from a prophylactic trial design where 2-wk-old (postnatal day 15) *mdx* or wild-type control mice received oral dosing for 6 wk with vehicle (cherry syrup), prednisolone (5 mg/kg), or vamorolone (15 mg/kg) as previously reported (23). The second “validation set” of diaphragm muscles was from an extended trial in older mice where *mdx* or wild-type mice were subjected to treadmill running to unmask mild phenotypes. Mice in this validation set were treated with either vehicle, prednisolone (5 mg/kg), or vamorolone (45 mg/kg) for 4 mo beginning at 2 mo of age (23). At the end point of each trial, diaphragm muscles were harvested and frozen in liquid nitrogen-cooled isopentane.

#### TaqMan miRNA low-density arrays.

We extracted total RNA from five diaphragm muscles per treatment group in the discovery set of samples from 8-wk-old mice. RNA was extracted using a modified TRIzol protocol with isopropanol precipitation at −20°C overnight. This RNA was reverse-transcribed to cDNA using a High Capacity cDNA Reverse Transcription Kit with RNase Inhibitor (Thermo Fisher, Carlsbad, CA), with miRNA-specific Megaplex RT Primers, Rodent Pools Set version 3.0 (Thermo Fisher). Levels of each of the miRNAs were profiled in the discovery set using TaqMan Array Rodent MiRNA A+B Cards Set v3.0 (Thermo Fisher).

#### Quantitative RT-PCR of individual miRNAs.

Specific miRNAs were quantified in the validation set of samples using individual TaqMan assays specific for each miRNA (Thermo Fisher) according to the manufacturer’s protocol. Assay IDs used include 000468, 001346, 002455, 001280, 000528, 002352, 000464, 002509, and 002248. Total RNA was converted to cDNA using multiplexed RT primers and High Capacity cDNA Reverse Transcription Kit (Thermo Fisher). The cDNA was preamplified using TaqMan PreAmp Master Mix (Thermo Fisher). The miRNAs were then quantified using individual TaqMan assays on an ABI QuantStudio 7 Real-Time PCR machine (Applied Biosystems, Foster City, CA).

#### Statistical analysis of miRNA expression.

In the discovery set experiments, levels of each miRNA were quantified in TaqMan low-density arrays (TLDAs) using Thermo Fisher Cloud software with the Relative Quantification Application (Thermo Fisher) tool. Data were analyzed by ANOVA with post hoc comparison of each group to the *mdx* vehicle-treated group. To identify focus miRNAs for study relating to drug efficacy, we selected all miRNAs that showed a significant difference for all three groups (wild-type, prednisolone, and vamorolone) compared with *mdx* vehicle. A *P* value of *P* ≤ 0.05 was set as the significance threshold, without adjustment for multiple comparisons. To reduce false-positive discovery in this setting, we used an evidence-based approach to identify efficacy-associated miRNAs where *1*) results from the multiple groups were cross-referenced, and *2*) all candidate miRNAs identified in this discovery set were then assayed in a separate validation set of mice. For the validation set experiments, we assayed levels of each individual miRNA in samples from a second, independent preclinical trial in adult *mdx* mice. Data from individual TaqMan Assays were quantified using QuantStudio Real-Time PCR version 1.3 software (Applied Biosystems). The levels of all miRNAs were normalized to the geometric mean of multiple control genes (60, 70). Comparison of groups was made by ANOVA with Holm-Sidak post hoc test comparing each group to *mdx* vehicle.

#### Bioinformatics.

We examined the regulation of each miRNA gene promoter to gain insight into the mechanisms of response to treatment. This was done by examining promoter binding by the inflammatory transcription factor nuclear factor-kappa B (NF-κB, or RELA) or by the GR (NR3C1) using chromatin immunoprecipitation sequencing (ChIP-seq) data. For both NF-κB and the GR, ChIP-seq data from the Encyclopedia of DNA Elements (ENCODE) were queried for physical binding to DNA loci encoding the human homologue of each miRNA target of interest (29, 41). In addition, we examined the following histone modifications, which are enriched at regulatory elements such as promoters or enhancers: histone H3K4 trimethylation (found near promoters), H3K4 monomethylation (found near regulatory elements), and H3K27 acetylation (found near active regulatory elements). For each of these analyses, we used UC Santa Cruz Genome Browser Release 4 (https://genome.ucsc.edu/index.html) with alignment to the GRCh37/hg19 genome build. Each ChIP-seq data set was analyzed using the ENCODE Regulation Super-Track listed under the Regulation menu. Binding by NF-κB or GR was assayed using the Txn Factor ChIP Track. In regions bound by each transcription factor, DNA motifs recognized by that transcription factor were identified through the Factorbook repository within this track. Consensus motif sequence logo pictograms for each transcription factor were also visualized through Factorbook. Histone modifications were examined using the Layered H3K4Me1, Layered H3K4Me3, and Layered H2K27Ac Tracks. Raw data images for visualization of gene loci and ChIP-seq data were obtained using the PDF/PS function in the View menu of the genome browser.

Binding by NF-κB was queried in ChIP-seq data sets produced using TNF-induced lymphocyte cell lines (GM10847, GM12878, GM12891, GM12892, GM15510, GM18505, GM18526, GM18951, GM19099, and GM19193) with ChIP-seq performed using an antibody to an NF-κB subunit (RELA). For the GR, we queried ChIP-seq data sets produced using dexamethasone-treated lung epithelial (A549) and endometrial (ECC-1) cell lines with ChIP-seq performed using an antibody to the GR (NR3C1). Histone modifications were queried in ChIP-seq data sets produced using lymphoblast (GM12878), stem (H1-hESC), myoblast (human skeletal muscle myoblasts), endothelial (human umbilical vein endothelial cells), lymphoblast (K562), keratinocyte (normal human epidermal keratinocyte), and lung fibroblast (normal human lung fibroblast) cell lines using antibodies specific to each histone modification.

To visualize behavior of miRNAs within individual mice for each group, we generated heat map images. Heat maps were generated using relative quantification values of TLDA data exported from the Relative Quantification Application of the Thermo Fisher Cloud software (Thermo Fisher) tool. Heat maps of miRNA expression were produced using Hierarchical Clustering Explorer Version 3.5 (http://www.cs.umd.edu/hcil/multi-cluster/) produced by the Human Computer Interaction Laboratory (University of Maryland, College Park, MD).

## RESULTS

### 

#### Discovery of miRNAome responses to muscular dystrophy disease and treatment.

To examine miRNA expression, we utilized TaqMan low-density quantitative PCR array cards to profile ~750 miRNAs within diaphragm muscle from a discovery set of mice. Samples for this experiment came from a trial that utilized a prophylactic approach (23). Briefly, *mdx* mice undergo a stage of peak severity characterized by widespread inflammation and necrosis from ~3–8 wk of age, followed by a recovery stage where mice show milder phenotypes (25). For this prophylactic preclinical trial, treatments were initiated in mice at postnatal day 15 to treat before and during the stage of peak severity. The discovery set of samples consisted of diaphragm from 8-wk-old untreated wild type (vehicle), untreated *mdx* (vehicle), prednisolone-treated *mdx* (5 mg/kg), and vamorolone-treated *mdx* (15 mg/kg) mice. Prednisolone was used here because it is the active form of prednisone, the current DMD standard of care. We chose to examine the diaphragm because respiratory function is important for DMD outcomes, because it is a severely affected muscle in *mdx* that is more comparable to DMD (65) and because diaphragm muscles are more evenly stressed between mice than purely voluntary muscles of the leg. Previously, in these same mice, we found that both prednisolone and vamorolone successfully improved grip strength, muscle pathology, and diaphragm inflammation (23). Additionally, we found in these same mice that prednisolone caused traditional steroid side effects of stunted growth, immunosuppression, and bone loss, whereas the dissociative steroid vamorolone successfully avoided these side effects. Here, using TLDA quantitative PCR array cards we detected ~500 miRNAs expressed in the diaphragm muscle from all groups.

We found that expression levels of 202 miRNAs showed a significant difference in at least one of the groups compared with the untreated *mdx* group (Supplemental Table S1; Supplemental Material for this article is available online at the Journal website). Comparing untreated *mdx* to wild-type mice, expression levels of 136 miRNAs were significantly different in dystrophic muscle. Treatment of *mdx* mice with the glucocorticoid prednisolone caused a significant change in 76 miRNAs in dystrophic muscle. In contrast, treatment with the dissociative steroid vamorolone only affected about half as many miRNAs, with expression of 41 miRNAs changed in *mdx* muscle.

**Fig. 1.**
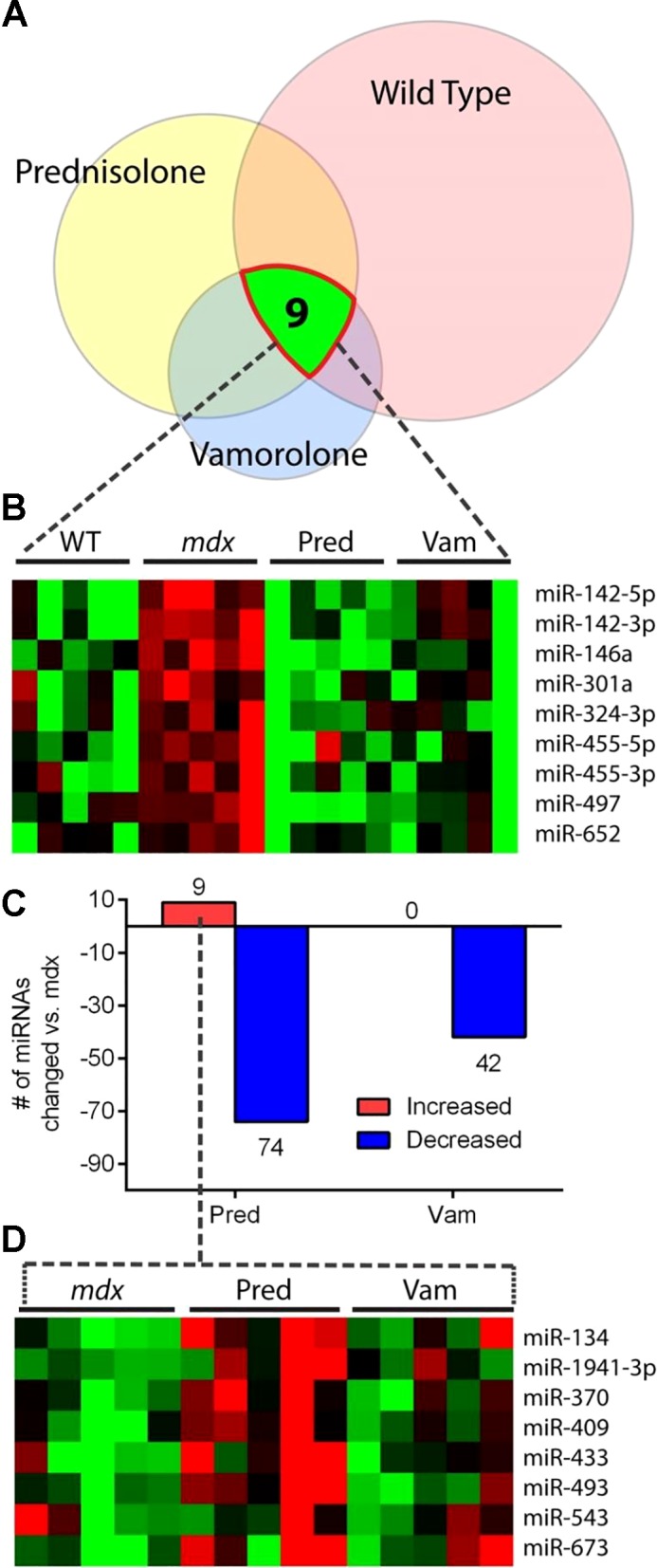
Summary of muscle miRNA changes discovered in response to dystrophy and its treatment. Expression of the miRNAome was quantified in diaphragm muscle of mice from a discovery set of mice (*n* = 5 mice per group). Groups included WT (vehicle), *mdx* (vehicle), *mdx* treated with prednisolone (5 mg/kg), and *mdx* treated with vamorolone (15 mg/kg), with mice treated from 2 to 8 wk of age in a prophylactic trial design. *A*: a Venn diagram illustrates the proportion of miRNAs that are significantly different than untreated *mdx* muscle in the WT, prednisolone, and vamorolone groups. The nine miRNAs that were significantly different in all three groups vs. untreated *mdx*, highlighted here, were chosen as a focus set of efficacy-associated miRNAs. *B*: heat map visualization of the expression of the nine efficacy associated miRNA markers within each individual mouse. *C*: bar graph showing the number of miRNAs that significantly increased or decreased in response to either of the drug treatments. *D*: heat map of the eight unique miRNAs that were increased by prednisolone. Heat map: red, increased; green, decreased. Pred, prednisolone; Vam, vamorolone; WT, wild type.

To identify a set of efficacy miRNA markers associated with both the muscular dystrophy disease process and a healthy response to treatment, we queried which miRNAs were different in all three groups (wild type, prednisolone, and vamorolone) compared with untreated *mdx* (Fig. 1). Using this approach, we identified a focus set of nine miRNAs (Fig. 1, *A* and *B*). All nine of these miRNAs were increased in muscular dystrophy and returned toward healthy wild-type levels as a result of treatment with both drugs (Table 1). Of these nine miRNAs, three have previously been found to be dysregulated in muscle disorders (miR-146a, miR-142-3p and miR-142-5p; 17, 28, 40). Interestingly, all nine of these miRNAs are involved in inflammatory signaling pathways and at least seven of them have been found to be upregulated in other inflammatory disorders (6, 9, 13–15, 18, 21, 28, 32, 34–36, 39, 40, 42, 44, 49–51, 59, 61, 67, 68, 71, 74, 75, 82); see Table 1 for miRNA-specific references).

**Table 1.** Nine miRNAs are elevated by dystrophy and respond to both drugs in the discovery set of diaphragm muscle

miRNA	WT, %	*Mdx*, %	Pred, %	Vam, %	miRNA Function Disease Associations	Sources
142-5p	43 ± 20**	100 ± 23	44 ± 11**	61 ± 23*	DC homeostasis; resolution of acute inflammation**/**IBD, Alzheimer’s, LGMD2D, DMD	14, 40, 44, 59, 75
142-3p	49 ± 19***	100 ± 10	50 ± 13***	65 ± 20**	Innate immunity; DC homeostasis; resolution of acute inflammation**/**SLE, LGMD2B/2D, DMD	18, 28, 44, 59
146a	56 ± 10***	100 ± 20	39 ± 6***	52 ± 12***	Stages inflammation**/**IBD, LGMD2A/2B, Myositis, MM, wasting, HF, MG, Alzheimer’s, MS	9, 15, 34, 36, 39, 49, 51, 68, 71
301a	81 ± 15*	100 ± 11	70 ± 18*	77 ± 12*	NF-κB positive feedback loop**/**IBD	21, 35
324-3p	75 ± 17*	100 ± 19	71 ± 14*	75 ± 13*	Induces and activates NF-κB**/**IBD	6, 13, 21
455-5p	61 ± 11*	100 ± 17	64 ± 30*	64 ± 15*	TGF-β Signaling**/**FSHD, LGMD2A, nemaline myopathy	15, 67
455-3p	63 ± 21*	100 ± 24	62 ± 18*	61 ± 14*	Innate immunity; cartilage development**/**IBD, Alzheimer’s	32, 50, 74, 82
497	72 ± 12*	100 ± 22	49 ± 11***	65 ± 11**	NF-κB feedback mechanism via IKKβ	42
652	66 ± 18*	100 ± 30	62 ± 14*	64 ± 18*	Inflammatory signals in immune cells	61

Values are expressed as % expression in comparison to *mdx* vehicle. DC, dendritic cell; DMD, Duchenne muscular dystrophy; FSHD, facioscapulohumeral muscular dystrophy; HF, heart failure; IBD, inflammatory bowel disease; LGMD2D, limb girdle muscular dystrophy type 2D; MG, myasthenia gravis; MM, Miyoshi myopathy; MS, multiple sclerosis; NF-κB, an inflammatory transcription factor named nuclear factor kappa-light-chain-enhancer of activated B cells; Pred, prednisolone; SLE, systemic sclerosis; TGF, transforming growth factor; Vam, vamorolone; WT, wild type.

* *P* ≤ 0.05, *P ≤ 0.005, ***P ≤ 0.0005.

ANOVA with post hoc comparison to *mdx* vehicle.

We next compared effects of the two drugs to examine consequences of their differing chemistries on genomic miRNA regulation and steroid side effects. Prednisolone, a traditional glucocorticoid, both activated and inhibited expression of miRNAs (Fig. 1, *C* and *D*). Treatment with prednisolone produced a significant increase in nine miRNAs (*P* ≤ 0.05), eight of which were unique. Interestingly, none of the miRNAs queried in the TLDA cards were significantly increased in response to vamorolone treatment. These data are consistent with the more selective dissociative chemistry of vamorolone, which can inhibit inflammatory signaling without activation of individual GR-regulated genes. Additionally, these data identify a set of prednisone-specific miRNAs whose activation is consistent with the activation of glucocorticoid side effects observed in these same mice.

#### Efficacy miRNA responses are conserved in an independent validation trial.

Having identified nine inflammatory miRNAs of interest in the discovery set of mice, we next sought to both validate the miRNA markers that we found and to expand upon our results to determine which ones are of utility in other disease stages or trial designs commonly studied in *mdx* literature (23, 25). To do this, we obtained a validation set of samples from a separate, independent trial using a different trial design characterized by a prolonged treatment regimen in older *mdx* mice. Samples were obtained at a trial end point of 6 mo. At this stage, *mdx* mice show less variability however they have gone through a recovery stage, which results in milder phenotypes that typically require treadmill exercise to unmask. Here, we used gene-specific quantitative RT-PCR to detect expression levels of each individual inflammatory miRNA within diaphragm muscle from the validation set. Differences in the trial design for the validation set of samples versus the previous discovery set include the age of mice (6 mo old), the stage of *mdx* disease (characterized by a phenotypic recovery and increased fibrosis), the added exercise protocol (treadmill running), the length of treatment (4 mo), and the dose (45 mg/kg) of vamorolone (23).

**Fig. 2.**
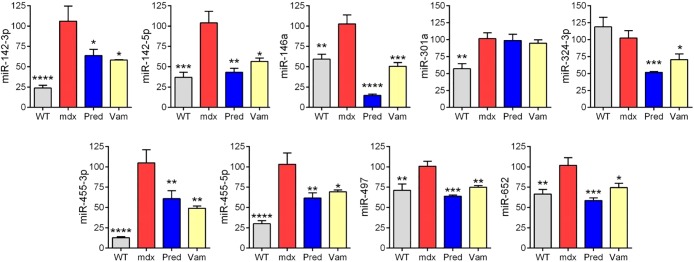
Behavior of efficacy miRNA signature is maintained in an independent validation trial. A validation set of samples was obtained from a second, independent *mdx* trial performed at a different stage of the *mdx* disease. Mice received daily oral vehicle, prednisolone (5 mg/kg), or vamorolone (45 mg/kg) for 4 mo, with treadmill running to unmask *mdx* phenotypes and muscle harvested at 6 mo of age. The nine miRNAs identified as associated with efficacy in the TLDA arrays were quantified in diaphragm muscle using quantitative RT-PCR in this second set of mice. (Values are graphed as % of untreated *mdx* expression levels; 1 outlier removed from miR-455-5p after significant Grubb’s test; *n* = 5 per group; ANOVA with post hoc comparison to *mdx* vehicle; *P ≤ 0.05, **P ≤ 0.01, ***P ≤ 0.0005). TLDA, Taqman low-density array.

Upon miRNA analysis of the validation set, we found all nine miRNAs showed a conserved response to disease and/or drug treatments (Fig. 2). One miRNA, miR-301a, showed a roughly twofold increase with disease (*P* = 0.002) but did not respond to drug treatment at this age. One other, miR-324-3p, showed a ~30%–50% decrease in response to both prednisolone (*P* = 0.0005) and vamorolone (*P* = 0.02) but did not show a difference between *mdx* and wild-type mice at this age. The other seven miRNAs all showed both a significant increase with muscular dystrophy (*P* ≤ 0.005), and a significant decrease toward healthy wild-type levels in response to both drugs (*P* ≤ 0.05). This confirmed that overall, inflammatory miRNAs can be reduced by vamorolone and prednisone at different stages of dystrophy.

#### Efficacy associated miRNAs indicate NF-κB inhibition mechanism.

**Fig. 3.**
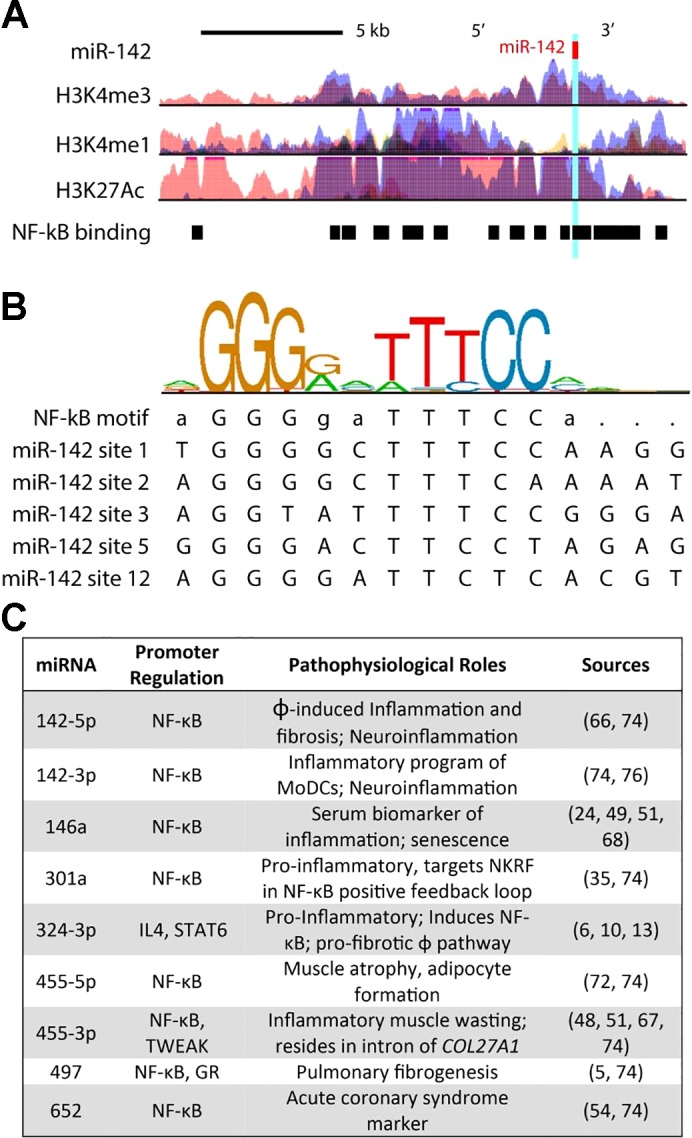
Promoter analysis of miRNAs indicates NF-κB signaling is a shared target of effective drugs. Transcription factor (NF-κB, GR) binding sites and histone (H3) modifications that mark regulatory regions were examined using ChIP-seq data from ENCODE. DNA-binding motifs for each transcription factor were identified through the Factorbook repository. *A*: schematic of the gene locus for miR-142, illustrating the binding site of 13 neighboring DNA loci that are bound directly by NF-κB. Corresponding epigenetic modification maps are provided showing the location of histone modifications associated with active promoters (H3K4me3) and poised/active enhancers (H3K4me1 and H3K27Ac) in the immediate vicinity of miR-142. *B*: sequence logo pictogram of base frequency at NF-κB binding sites, with the consensus NF-κB motif provided immediately below. Also provided are five representative NF-κB binding site sequences near miR-142, listed in order from the 5′ to 3′ direction. *C*: summary of promoter analysis and literature data indicating each miRNA and known factors or conditions associated with its transcriptional regulation. ChIP-seq, chromatin immunoprecipitation sequencing; COL27A1, collagen type 27 alpha 1 chain; ENCODE, Encyclopedia of DNA Elements; GR, glucocorticoid receptor; IL4, interleukin 4; MoDCs, monocyte-induced dendritic cells; φ, macrophage; NKRF, NF-κB-repressing factor; STAT6, signal transducer and activator of transcription 6; TWEAK, TNF-like weak inducer of apoptosis.

We queried both NF-κB and GR transcription factor ChIP-seq data from ENCODE and the established literature to gain insight into the transcriptional regulation of each miRNA (Fig. 3). We found that the DNA promoters of eight out of the nine identified miRNAs contain one or more DNA sites that are bound by the inflammatory transcription factor NF-κB (74). This is supported by previous reports demonstrating NF-κB-specific regulation of miR-146a (68), miR-301a (35), and miR-455-3p (48). The other miRNA, miR-324-3p, is regulated by signal transducer and activator of transcription 6 and in turn activates NF-κB, thereby participating in inflammatory NF-κB signaling as well (13). In contrast, only one miRNA, miR-497, had a DNA promoter site bound by the GR; however, it also possessed an NF-κB binding site with its promoter region (Fig. 3). In addition to being regulated by NF-κB, when inappropriately expressed these miRNAs are associated with chronic inflammation, muscle wasting, fibrosis, and adipocyte formation (5, 6, 10, 24, 35, 49, 51, 54, 66, 67, 72, 76; refer to Fig. 3).

One of the most interesting miRNAs in the ChIP-seq analysis was miR-142, which we found is in a DNA locus surrounded by at least 13 DNA elements bound directly by NF-κB (Fig. 3, *A* and *B*). All 13 of these NF-κB binding sites overlapped with corresponding ChIP-seq data that detect histone modifications, which correspond to active regions of transcription regulation (H3K4me3, H3K27Ac, and H3K4me1). Together, these data indicate that the upregulation of NF-κB-activated miRNAs is a signature of dystrophic muscle and that inhibition of chronic NF-κB signaling is a signature shared by two distinctly different but effective steroidal drugs.

#### Prednisone activates miRNAs associated with side effects and the 14q32 locus.

**Fig. 4.**
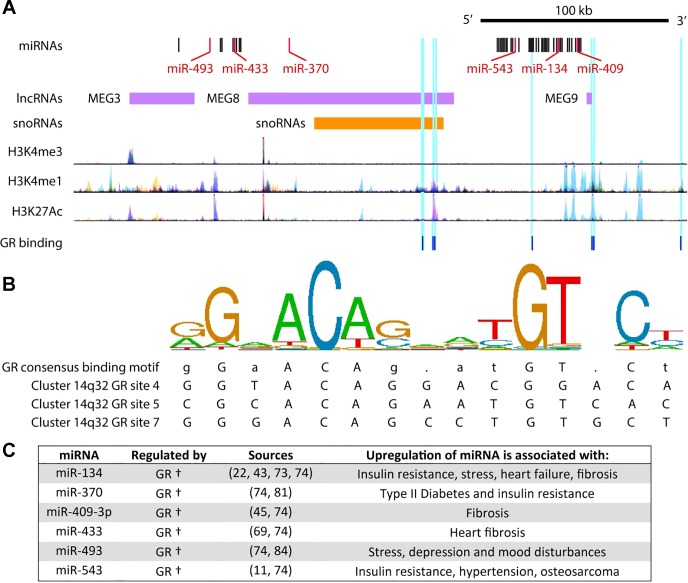
Prednisolone increases miRNAs associated with GR regulation and the 14q32 mega cluster. For the conserved miRNAs that were specifically elevated in prednisolone-treated mouse muscle, we analyzed the genomic loci, transcription factor binding sites (GR and NF-κB), and histone (H3) modifications using ChIP-seq data from ENCODE. DNA motifs bound by the GR were identified through Factorbook. *A*: all six of the miRNAs are transcribed from a well-conserved noncoding RNA cluster (mouse 12F1) which is extensively characterized in humans at the 14q32 locus; that human cluster is depicted here along with corresponding GR-binding sites, as well as histone modifications that correspond to active gene promoters (H3K4me3) and poised or active gene enhancer elements (H3K4me1, H3K27Ac). No NF-κB-binding sites are found at this locus. *B*: sequence logo pictogram of base frequency at GR binding sites, with the consensus GR motif sequence provided immediately below. Also provided are three representative GR-binding site sequences from this locus numbered from the 5′ to 3′ direction. *C*: summary of bioinformatic analyses for each miRNA, with a list of conditions that are associated with increased levels of each miRNA. †encoded by the 14q32 cluster of miRNAs with GR-bound enhancers. ChIP-seq, chromatin immunoprecipitation sequencing; ENCODE, Encyclopedia of DNA Elements; GR, glucocorticoid receptor; lncRNA, long noncoding RNA; MEG, maternally expressed gene; snoRNA, small nucleolar RNA.

Next, we queried ChIP-seq data and established literature to gain insight into the set of miRNAs that we found were specifically activated by prednisolone (Fig. 4). Here, we focused on the six miRNAs conserved between mice and humans and on pathways relevant to DMD or steroids. Interestingly, we found that all six of these miRNAs (miR-134, miR-370, miR-409, miR-433, miR-493, miR-543) are transcribed from the same miRNA cluster on mouse chromosome 12F1. This locus is well conserved and has been extensively documented in humans where it resides on chromosome 14q32 (63). Our analysis thus focused specifically on the homologous human cluster (Fig. 4, *A* and *B*). We found that this 14q32 cluster contains seven GR-DNA binding sites and is devoid of NF-κB binding sites (74). At least six of these GR binding sites corresponded to active regulatory enhancer elements. miR-543 has been previously shown to be upregulated by pharmacological glucocorticoids (11). This locus has also been observed to be upregulated by acute stress consistent with upregulation of cortisol, the body’s natural glucocorticoid (43). Examining the functional roles of miRNAs upregulated by prednisolone, we found that all are upregulated in states consistent with known side effects of prednisolone treatment. These include insulin resistance (27, 73, 81), behavior changes (43, 81), fibrosis (45, 69), increased risk for heart failure (22, 77), stress (43, 84), and hypertension (11). Together, these data indicate that prednisone specifically activated a separate set of miRNAs in a manner that is consistent with the negative side effects of currently prescribed steroids.

## DISCUSSION

**Fig. 5.**
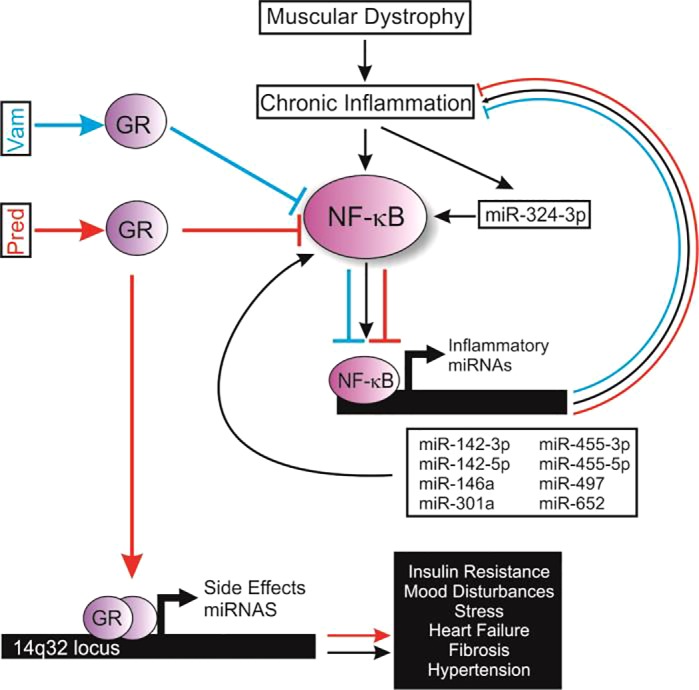
Proposed model of NF-κB and GR-regulated miRNAs in the treatment of muscular dystrophy. In DMD, inflammatory signaling promotes the chronic activation of NF-κB. This, in turn, activates NF-κB gene targets, including miRNAs that regulate the expression of proteins in the NF-κB signaling pathway, creating a chronic inflammatory feedback loop. Here we show that the NF-κB-regulated miRNAs miR-142-3p, miR-142-5p, miR-146a, miR-301a, miR-455-3p, miR-455-5p, miR-497, and miR-652 are all elevated in dystrophic muscle. These NF-κB-regulated miRNAs are all effectively decreased by both vamorolone (Vam) and prednisone (Pred) treatment, via the GR. miR-324-3p, a miRNA that activates NF-κB in a positive feedback loop, is also decreased by both drugs. Acting through a separate pathway which can be selectively avoided by dissociative steroid chemistries, prednisone also directly causes GR-mediated transactivation of gene transcription. This results in elevated levels of a miRNA cluster located on chromosome 14q32. These microRNAs are associated with steroid side effects such as insulin resistance, hypertension, stress, and mood disturbances. Blue lines, pathways affected by vamorolone; red lines, pathways affected by prednisolone. DMD, Duchenne muscular dystrophy; GR, glucocorticoid receptor.

Our data identify a set of miRNAs that are elevated by dystrophic disease and that respond to treatment with both prednisolone and the dissociative steroid vamorolone. Of the nine miRNAs we identified, three have been previously found to be elevated in DMD or *mdx* muscle (miR-146a, miR-142-3p, and miR-142-5p), whereas the elevated expression of six others in dystrophic muscle are novel. Analyzing the regulation and functions of these nine miRNAs reveals that all nine are involved in proinflammatory signaling. By comparing prednisone and vamorolone, we see that these two effective but distinctly different GR ligands share efficacy as anti-inflammatory drugs that downregulate this network of inflammatory miRNAs. In contrast, vamorolone, unlike prednisolone, avoids off-target activation of miRNA transcription associated with negative steroid side effects, such as insulin resistance, adrenal suppression, hypertension, and behavior issues. This is consistent with both preclinical *mdx* mouse and recently completed human Phase I trials, which show vamorolone avoids or has substantially reduced steroidal side effects in comparison to prednisone (23, 26). Together, our work identifies a network of miRNAs associated with chronic inflammation and validates NF-κB signaling as an in vivo target of efficacious dissociative steroids (see Fig. 5 for model).

miRNAs fine tune gene expression in a multitude of signaling pathways and cellular processes. Similarly, miRNAs play a key regulatory role in the core inflammatory signaling pathway driven by NF-κB. This pathway is inappropriately upregulated in many inflammatory disorders and drives chronic muscle inflammation in DMD (7, 47). Of the miRNAs we describe here, eight are directly regulated by NF-κB (6, 13, 35, 48, 51, 74). Some, in turn, also regulate the initiation and resolution of the inflammatory response by targeting other key factors in the NF-κB signaling pathway. Thus, understanding the mechanisms governing miRNA dysregulation in relation to NF-κB signaling is particularly relevant to anti-inflammatory drug development in DMD. We will discuss some of the miRNAs of note below.

miR-142 is dysregulated across multiple diseases and may provide a novel therapeutic target. Here, we find both mature miR-142 family members (miR-142-3p, miR-142-5p) are elevated with dystrophic disease and their expression is quelled by both prednisolone and vamorolone treatment. miR-142-3p is highly expressed in monocytes (18) and lymphocytes (30), suggesting it is a marker of inflammatory infiltration. Corroborating our data here, another report observes elevated miR-142-3p levels in the gastrocnemius of three distinct muscular dystrophy model mice: *Dysferlin-null*, *α−sarcoglycan* (*Sgca*)*-null*, and *mdx* (28). This same report finds when *Sgca* is reexpressed in *Sgca-null* mice, miR-142-3p levels move toward their wild-type counterparts (28). Not surprisingly, studies focusing on miR-142-5p show similar findings. One report shows miR-142-5p is increased in single muscle fibers isolated from *mdx* and *Sgca-null* muscles and is induced in response to acute injury in *mdx* (40). Of particular interest, a recent study in mouse models of experimental colitis finds that inhibition of miR-142-5p is effective at improving disease outcomes (14). In this colitis study, the most important disease feature that improves with miR-142-5p inhibition is muscle wasting, which is known to be regulated by NF-κB (2). Together, these findings suggest miR-142 family members can serve as biomarkers of drug efficacy across different muscular dystrophies. Furthermore, the ubiquitous nature of miR-142 dysregulation across a multitude of inflammatory disorders makes these miRNAs attractive as potential therapeutic targets in states of chronic inflammation.

miR-146a is one of the most prevalent miRNAs that appears in the literature in instances of chronic inflammatory disorders (9, 15, 34, 36, 39, 49, 51, 71). miR-146a is highly associated with inflammation, is induced by NF-κB in immune cells (68), and is also expressed directly in muscle (17). Acute miR-146a activation dampens NF-κB-mediated inflammation (68); however, prolonged induction of miR-146a exacerbates inflammation (19, 37). In diseases where chronic inflammation is present, miR-146a levels are highly elevated both in the serum as well as in tissues affected by disease (15, 34, 36, 39, 49, 51, 71). Previously, we reported that miR-146a serum levels predict patient response to anti-inflammatory (prednisone, Remicade) treatment in inflammatory bowel disease (IBD; 24). In a separate report, we showed that miR-146a participates in a muscular dystrophy feedback loop wherein it specifically inhibits the production of dystrophin in Becker muscular dystrophy and in a mouse model of DMD exon skipping (17). Our current findings strengthen claims that miR-146a is both a promising therapeutic target and a pharmacodynamic biomarker.

Previous reports describe a role for miR-455 family members in immune signaling and muscle wasting. The transcription of miR-455-3p is controlled by NF-κB in macrophages (48). A report by Eisenberg et al. (15) shows increased miR-455-5p in muscle biopsies from patients with facioscapulohumeral muscular dystrophy, limb girdle muscular dystrophy 2A and nemaline myopathy. miR-455-3p is encoded within the intron of *COL27A1*, a gene that encodes a cartilage collagen (67). Expression of miR-455 is induced by TNF-like weak inducer of apoptosis, which plays a key role in skeletal muscle wasting (51), and miR-455-5p is implicated in skeletal muscle atrophy (72). Interestingly, miR-455-3p is also implicated in Alzheimer’s disease (32). Together, these data suggest miR-455 family members may function as a potential marker of inflammation, atrophy, and drug efficacy.

Three miRNAs found in our study specifically regulate the duration and extent of NF-κB signaling via feedback mechanisms. miR-301a was reported to be the most potent activator of NF-κB out of hundreds of miRNAs, and it exerts its actions via downregulation of the NF-κB repressing factor (35). The miR-301a promoter contains an NF-κB DNA consensus element, allowing a positive feedback mechanism of NF-κB signaling: miR-301a represses NF-κB repressing factor, in turn promoting NF-κB activation, which activates miR-301a transcription. Another identified miRNA, miR-324-3p, functions in a transcription factor-like manner to trigger NF-κB transcription via sequence-specific promoter binding (13). Elevated miR-324-3p is also observed in IBD (52) and is rapidly induced after focal cerebral ischemia (12), further implicating this miRNA in driving inflammatory processes. miR-497 transcription is driven by NF-κB (42). In cases of acute inflammation, miR-497 participates in a feedback mechanism by targeting IKKβ, a kinase required for NF-κB activation (42). miR-497 is associated with regenerative capacity of muscle stem cells (62) suggesting it is transcribed both in immune cells and skeletal muscle. The above reports describing miRNA involvement in NF-κB -mediated feedback focus specifically on acute inflammation; the consequences of chronic miR-301a/miR-324-3p/miR-497 overexpression on NF-κB signaling have not been documented. Our data, however, suggest that persistent expression of these miRNAs drives a feed-forward loop of prolonged NF-κB activation and inflammation; this can be effectively attenuated by prednisolone and vamorolone treatment.

For the first time, we find that expression of a noncoding RNA cluster in the genome is increased by prednisone treatment of dystrophic muscle. Specifically, we identify six miRNAs within this cluster that appear to be coregulated and located by GR-bound enhancer elements. This cluster is well conserved across mammalian species and is among the largest polycistronic clusters. It is best characterized in humans where this cluster is on chromosome 14q32 and encodes 54 miRNAs (63). All six of the miRNAs increased from this locus are also known to be elevated in conditions that are consistent with prednisone side effects, including insulin resistance (27, 76, 81), mood disturbances (43, 81), stress (43, 84), and hypertension (11). Because prednisolone increases *mdx* heart fibrosis (9) and heart failure is a leading cause of death in DMD, increases here in miR-433 and miR-134 are interesting as they are a regulator of heart fibrosis and a serum biomarker indicative of increased risk of heart failure, respectively (22, 69). In addition to miRNAs, the 14q32 locus encodes two long noncoding RNAs, which also may have functions that are relevant to DMD and its treatment with steroids. These two long noncoding RNAs are maternally expressed gene (MEG) 8 and MEG3. MEG8 is preferentially expressed in skeletal muscle and is increased in muscle hypertrophy as observed in callipyge (or “beautiful buttocks”) sheep (4). MEG3 expression is enriched in cardiac fibroblasts and intriguingly, the inhibition of MEG3 prevents heart fibrosis and diastolic dysfunction via regulation of matrix metalloproteinase 2 in a mouse model of heart damage (53). Moving forward, it will be interesting to determine the full extent of upregulation at the 14q32 locus upon prednisone treatment and the health impact that this may have on muscular dystrophy patients.

It is interesting to note that there were many similarities between dysregulated miRNAs reported here in muscular dystrophy and what has been reported in other inflammatory diseases, in particular for IBD. Specifically, in IBD six of the nine miRNAs reported here (miR-142-3p, miR-142-5p, miR-146a, miR-342-3p, miR-301a, miR-455-3p) are elevated in the serum and intestinal mucosa of patients with IBD and mouse models of IBD (3, 16, 21, 50). This suggests that the identified set of miRNAs reported here reflect a general signature of chronic inflammation and disease. Given that many of these miRNAs have been identified in inflammatory cells and processes, it is possible that these shared miRNA signatures are representative of inappropriate crosstalk between immune cells and the diseased tissue microenvironment. Moving forward, it will be important to identify and understand the mechanisms that drive inappropriate immune cell-tissue crosstalk and identify targets that could dampen these signals.

One of the miRNAs we identified in our discovery set of samples, miR-301a, did increase with disease but did not show a response to either drug in the validation set of samples. This may be due to a switch in its transcriptional control as the *mdx* disease transitions from a younger and more inflammatory stage, to an older and more fibrotic stage. In addition to NF-κB, the transcriptional promoter of miR-301a is affected by pathways that increase with fibrosis and with age, via transcription factors within transforming growth factor-β (TGF-β) (7, 55, 56, 74, 83) and β-catenin (78, 79) pathways, respectively. Because fibrosis increases with *mdx* age, particularly in the diaphragm, a shift to combinatorial control of miR-301a expression by TGF-β and/or β-catenin pathway transcription factors could circumvent the effects of GR ligands on NF-κB. Because miR-301a increases NF-κB signaling and in some instances appears to avoid inhibition by GR ligands, it will be interesting to study this miRNA further, as inhibitors of it could provide a mechanism to enhance anti-inflammatory efficacy further through a cotherapy strategy.

There is growing enthusiasm with regards to targeting miRNAs as therapeutic agents via antisense technology. There are currently nine miRNA therapeutics that are in preclinical or clinical development, reviewed in (8). We suggest the miRNAs described here may be a defining signature of chronic inflammation and inappropriate immune cell-muscle cross talk. These findings provide a rationale for the development of miRNA inhibition agents as a potential strategy to treat diseases of chronic inflammation.

In broader terms, this work highlights muscular dystrophy as a good scientific system to provide insights into chronic inflammation pathways relevant to a much larger group of disorders. Elevated NF-κB signaling is present in dystrophic muscle even in infants with DMD, years before the onset of symptoms (7). We find that prednisone, the DMD standard of care and one of the most widely prescribed drugs in the world, shares efficacy with a more selective steroid by inhibiting this chronically elevated NF-κB signaling in *mdx* mice. Here we identify nine miRNAs that appear to largely behave as a set, all increasing with dystrophy and responding to treatment with drugs that share NF-κB inhibition as a mechanism of action. Interestingly, the majority of these miRNAs appear to be conserved across diseases, as others have observed them to be elevated in IBD and other diseases with chronic inflammation (14, 16, 35, 38, 52, 67). We find that at least one of these miRNAs, miR-146a, shows conserved behavior across species with muscular dystrophy (mouse, dog, and human) (17). We also find that miR-146a shows conserved drug responses between tissue and serum (47), providing a noninvasive serum biomarker that responds to both disease and treatment. Moving forward, it will be important to determine if the other miRNAs here can provide serum biomarkers as well, and if these miRNAs can be targeted as a next-generation approach to treat diseases of chronic inflammation.

## GRANTS

This work was funded by the Foundation to Eradicate Duchenne, the Clark Charitable Foundation, and the National Institutes of Health. A. Fiorillo is funded by the Department of Defense (W81XWH-17-1-047) and the NIH (1L40-AR-070539-01). C. Heier is funded by the NIH (R00-HL-130035, U54-HD-090254, L40-AR-068727). Both A. Fiorillo and C. Heier are additionally funded by the Clark Charitable Foundation and the Foundation to Eradicate Duchenne.

## DISCLOSURES

A. Fiorillo, C. Tully, and C. Heier have no conflict of interest relevant to this study. J. Damsker is employed by and has stock options in ReveraGen BioPharma, Inc., which owns intellectual property relating to vamorolone. K. Nagaraju and E. Hoffman have founder shares and a board membership with ReveraGen BioPharma, Inc.

## AUTHOR CONTRIBUTIONS

A.A.F., J.M.D., K.N., E.P.H., and C.R.H. conceived and designed research; A.A.F., C.B.T., and C.R.H. interpreted results of experiments; A.A.F., C.B.T., and C.R.H. analyzed data; C.B.T. and C.R.H. performed experiments; A.A.F. and C.R.H. prepared figures; A.A.F. and C.R.H. drafted manuscript; A.A.F., K.N., E.P.H., and C.R.H. edited and revised manuscript; A.A.F., E.P.H., and C.R.H. approved final version of manuscript.

## Supplemental Data

Table 1 Table 1 (.xlsx 38 KB)
